# Evaluating the feasibility and uptake of a community-led HIV testing and multi-disease health campaign in rural Uganda

**DOI:** 10.7448/IAS.20.1.21514

**Published:** 2017-03-30

**Authors:** Jane Kabami, Gabriel Chamie, Dalsone Kwarisiima, Edith Biira, Peter Ssebutinde, Maya Petersen, Edwin D. Charlebois, Moses R. Kamya, Diane V. Havlir, Tamara D. Clark

**Affiliations:** ^a^ Department of Medicine, Makerere University-University of California, San Francisco Research Collaboration, Kampala, Uganda; ^b^ Division of HIV, Infectious Diseases and Global Medicine, University of California, San Francisco, CA, USA; ^c^ Department of Health, Bwizibwera Health Centre IV, Uganda Ministry of Health, Mbarara, Uganda; ^d^ Berkeley School of Public Health, University of California, Berkeley, CA, USA; ^e^ Center for AIDS Prevention Studies, University of California, San Francisco, CA, USA; ^f^ Department of Medicine, School of Medicine, Makerere University College of Health Sciences, Kampala, Uganda

**Keywords:** HIV testing, community health campaign, mobile testing, hypertension, diabetes, Africa

## Abstract

**Introduction:** Multi-disease community health campaigns can be effective for population-wide HIV testing in a research setting (SEARCH: NCT01864603). We sought to evaluate feasibility and uptake of a community-led health campaign (CLHC) planned and implemented by village leaders and local clinic workers in Uganda.

**Methods:** Over five months in 2014, locally elected village leaders and Ministry of Health (MoH) clinic staff in a rural parish in Uganda planned a census followed by a CLHC, after training by two SEARCH trial consultants and by leaders from a neighbouring parish that had previously participated in a SEARCH health campaign. We defined feasibility as: (1) elected leaders’ participation in training and implementation of pre-campaign census and mobilization activities; (2) implementation of all campaign activities by MoH-funded, local clinic staff; and (3) community participation in the campaign, including point-of-care screening for HIV, malaria, hypertension and diabetes, and same-day referral for male circumcision and family planning (FP). Costing of all salaries and supplies was conducted.

**Results:** Elected leaders from all eight villages in the parish participated in CLHC training. They and local clinic staff met monthly to select and plan CLHC services. Village leaders then leveraged existing volunteer health teams to perform a door-to-door census, enumerating 5,202 parish residents over 2 weeks. 2,753 (53%) residents participated in the 6-day CLHC. Of 1,584 adult participants, 1,474 (93%) tested for HIV: 105/1,474 (7.1%) tested HIV positive. 27% (751/2,753) of participants reported fever and underwent malaria rapid diagnostic testing: 5.3% (40/751) tested positive. Among adults screened, 19% (271/1,452) were hypertensive, and 3% (18/637) had a random blood sugar >11.1 mmol/L. Of 805 men and boys (>10 years), 91 (11%) accepted same-day clinic referral and underwent medical circumcision. Of 900 women offered same-day long-term FP referrals, 25 accepted. The CLHC cost, including census, mobilization and testing services, was $23,597 ($8.57/participant).

C**onclusions:** Elected village leaders successfully planned and conducted a 6-day multi-disease health campaign with service provision by local clinic staff that reached over half of a rural Ugandan community. These data suggest it is feasible for local leaders and clinics to adopt a multi-disease health campaign approach to scale-up HIV testing in rural Africa.

## Introduction

Increasing HIV testing coverage remains a challenge for most of sub-Saharan Africa, where less than half of HIV-infected persons are estimated to know their HIV status [1]. To achieve the recent UNAIDS goal that 90% of all people living with HIV know their HIV status by 2020, broad implementation of evidence-based strategies to rapidly scale-up HIV testing is necessary [1]. Integrating testing approaches that studies have demonstrated to be effective into existing health programs and achieving community acceptance from local leaders and community members, will be critical to the success of implementing population-wide HIV testing across a variety of African settings.

Multi-disease community health campaigns are an effective way to rapidly increase HIV testing coverage in rural east Africa [2–4], as demonstrated by the Sustainable East Africa Research on Community Health collaboration (SEARCH; NCT: 01864603) [5–7]. Community health campaigns (CHC) offer several potential advantages over other approaches to HIV testing. They can serve as a platform for other public health priorities such as non-communicable disease screening, they may normalize HIV testing as part of routine health delivery, thereby reducing stigma, and they decentralize services to convenient community sites, reducing transport costs as a barrier to accessing care [7]. However, data are lacking on the feasibility of transitioning from research-led health campaigns to fully community-led multi-disease health campaigns.

In Uganda, Ministry of Health (MoH) policy recommends health promotion through empowerment of local communities to participate in decision-making and planning for local health service provision [8,9]. On a village level in rural Uganda, locally elected leaders, with support from volunteer health teams (VHTs), play an important role in health promotion by improving access to care and acting as a bridge between villagers and clinics. We sought to evaluate the feasibility of having local elected village leaders and Ministry of Health (MoH) clinic staff in a rural Ugandan community plan and carry out a multi-disease health campaign, with consultation and support from the SEARCH trial, and to evaluate community member uptake of services at the campaign.

## Methods

### Campaign setting and pre-existing health services

Rwebishekye Parish in Mbarara District in south-western Uganda is a rural agricultural community composed of eight villages and an estimated 6,400 residents based on 2002 Uganda National Census projections for 2012 [10]. Bwizibwera Health Centre IV (BWB HCIV), the primary government health facility for Rwebishekye residents, is located approximately 5 kilometres from the farthest village. BWB HCIV has two medical officers (physicians), two clinical officers and a team of midwives and nurses who offer diagnostic and treatment services for HIV (including antiretroviral therapy), malaria, and TB, as well as for hypertension and diabetes (i.e. anti-hypertensives, such as hydrochlorothiazide and nifedipine, and oral diabetic treatments such as metformin) in the “NCD” clinic. The same team of health workers and support staff also provide antenatal care, acute medical, emergency obstetric care and surgical services. The Health Centre provides clinical services free of charge to patients.

### Feasibility

We defined feasibility of the community-led health campaign as: (1) elected leaders’ acceptance and participation in campaign training, and implementation of pre-campaign census and mobilization activities; (2) implementation of all campaign activities by local MoH-funded clinic staff. This definition of feasibility focuses on aspects of health campaign implementation by locally elected leaders (e.g. campaign training, and census implementation), and by local clinic staff (e.g. health service delivery at the campaign) that have previously been staffed by research assistants in the SEARCH trial, in order to provide data on how to transition from research-led to community-led campaigns.

### Uptake

We defined uptake as census-enumerated resident participation in campaign activities, including point-of-care screening for HIV, malaria, hypertension and diabetes, and same-day referral for safe male circumcision and family planning services to parish residents.

### Elected leader training

Elected village leaders and local MoH leadership were approached by two consultants from the SEARCH Trial and from elected village leaders from a neighbouring parish that had previously participated in a SEARCH health campaign, and invited to receive training over three days on implementation of community census and pre-campaign mobilization activities. Training included review of how health campaigns are organized (including participant “flow” through the campaign stations), how to create and use census registers, and how to record campaign participant information. Elected leaders held three meetings with local MoH-funded clinic staff to plan, select and agree on campaign health services based on local health priorities.

### Community mobilization

Elected leaders designed and implemented community mobilization activities, including community meetings, announcements at public gatherings, and the distribution of posters and flyers in the 5 months before the campaign from April to August 2014. Village leaders selected three well known and centrally located sites across the parish for the campaigns, so as to maximize participation and reduce participants’ transport costs to the campaign.

### Baseline census

Over 2 weeks in August 2014, elected village leaders worked with existing local volunteer health teams (VHTs: volunteers from the community who support health promotion activities) to perform door-to-door enumeration of all residents living in their assigned villages. If no adult resident was home during an initial census visit, two additional visit attempts were made (three visits total), in an attempt to reach all residents. The census allowed the leaders to obtain an estimate of the baseline population size and of testing coverage at the campaign. Census workers used a simple register to record basic information about household members such as age, sex, marital status, and occupation. Identification numbers were created and assigned to all residents during census enumeration. Campaign workers then used resident name, gender, age, and village to identify and verify campaign participation.

### Community-led health campaign (CLHC)

Elected village leaders and VHTs worked directly with MoH clinic staff to choose the services provided at the CLHC based on local health priorities and local clinic resources, and designed the CLHC in consultation with advisors from the SEARCH trial and leaders in the neighbouring community of Kakyerere Parish who had experience in conducting CHCs, as previously described [6,7]. Local MoH clinic staff provided confidential health service delivery throughout the campaign. Registers from census enumeration were used to verify the identity of campaign participants, and to identify non-residents, with confirmation by locally elected leaders. During the campaign, elected leaders assisted only with identification of community residents from census registers at the registration (welcome) area and efficient participant flow through the campaign, but did not participate in health service delivery. Campaign participants who came from neighbouring communities were allowed to participate in the campaign, but were not included in the analysis. During the campaign, two SEARCH trial consultants provided limited technical assistance on mobilization, supplies management and CLHC staff training.

Elected leaders and MoH clinic staff selected the following services for the CLHC: screening for HIV, hypertension, diabetes and malaria, male condom distribution, referral for medical circumcision for men, and family planning services for women. Both medical male circumcision and family planning referral services were not initially presented as options by the SEARCH trial consultants, but rather added on the suggestion of MoH clinic staff during planning meetings. Over a period of six consecutive days (for 8–10 h/day), MoH clinic staff at the campaign performed rapid point-of-care screening for HIV following Uganda MoH guidelines [8], diabetes screening [11] and malaria testing, all with a single finger-prick blood collection. Elected leaders chose public places with sufficient space, and based on convenience and central location, as campaign sites, and used tents to provide protection from sun and rain to staff and participants. Services were offered based on participant age. Adult participants (≥15 years) were screened for HIV, diabetes and hypertension, regardless of their prior testing history or self-reported HIV status. All participants screening positive for HIV, diabetes and hypertension were given an appointment at the local clinic within one month of the campaign. Participants of any age with self- (or parent-) reported fever at the campaign were tested for malaria by rapid diagnostic test (RDT). All participants with positive malaria RDTs were provided treatment at the CLHC during one-on-one post-test counselling by MoH clinic staff. All children from 6 months to 5 years of age received vitamin A supplementation. Mebendazole tablets were provided for deworming to children from 1 to 5 years of age. All men or boys >10 years of age who attended the CLHC were offered information on safe male circumcision (SMC) and those who consented were transported from the CLHC to the local clinic for same-day circumcision. Boys between 11 and 17 years needed additional parent/guardian assent before referral for SMC. Women of reproductive age received family planning counselling regarding short- and long-term family planning methods, and were offered same-day clinic referral for long-term family planning methods of their choice.

Services at the CL-CH were provided in the following order: after initial registration, participants were first directed to a “Vital signs station” for measurement of blood pressure, before accessing the CL-HC laboratory for finger-prick blood testing (HIV, diabetes and malaria testing). Additional services for which referrals were made (e.g. circumcision, family planning, or for persons screening positive for a diagnostic test), took place during one-on-one post-test counselling, prior to the CL-HC exit station. At the exit station, Vitamin A supplementation and de-worming treatment services were provided, male condoms distributed to adults, and a raffle took place for those who completed all campaign activities, in which campaign participants could win prizes. The total value of all raffle prizes distributed was US $500.

### Statistical and costing methods

Measures of prevalence of each disease were estimated using the sample proportion with the total number of participants screened as the denominator. Stata version 11 software (Texas, USA) was used for analysis.

All census and campaign expenses were identified through extraction of cost information from expenditure records and study logs, supplemented by interviews with SEARCH consultants and MoH clinic staff that delivered the services. For the elected leaders and volunteer health teams, we calculated the cost of six days of daily transport reimbursements to reach CL-HC sites. For the campaign, the costing methods focused on resources utilized and economic costs, rather than out-of-pocket costs. We classified the resources under four main categories: personnel (including fringe benefits), recurring supplies and goods, recurring services (i.e. transportation, fuel, catered staff lunch), and capital goods and equipment. Personnel costs included a daily per diem for MoH workers during the six campaign days to cover off-site work. Costs of capital items were amortized on a straight-line basis over three years for lab equipment and 5 years for furniture, vehicles and computers, assuming no salvage value. The other activity costs also included overhead and administrative costs, training (including consultant costs for each day of training), community mobilization (including the cost of poster/flyer printing and distribution), transportation (including transport reimbursements during the census and campaign days), counselling, and laboratory-testing costs for all diseases screened. All costs were converted to US dollars based on the 2014 exchange rate of 2,463 Ugandan shillings per US dollar.

For a scaled-up replication model, we projected costs using MoH facilities, capital goods and government staff average salaries, without SEARCH trial support. Modified costs include using existing school or health centre facilities infrastructure rather than SEARCH-trial infrastructure (e.g. canvas tents, tables and chairs purchased for campaign activities); using MoH salaried coordinators and laboratory technicians; excluding SEARCH-supported expenses (e.g. reimbursement of transport costs for staff attending training meetings, and T-shirts provided to staff conducting the CLHC).

### Ethical consideration

The Makerere University School of Medicine Research and Ethics Committee and the Ugandan National Council on Science and Technology (Uganda), and the University of California, San Francisco Committee on Human Research approved the consent procedures and the study. All participants, or their parent/guardian, provided verbal informed consent in their preferred language. Children from 13–17 years could provide verbal consent if a parent/guardian was not present, whereas children <13 years old could not participate without an assenting parent/guardian present, in accordance with Uganda Ministry of Health guidelines [8].

## Results

### Feasibility and acceptability of training

All locally elected leaders from the eight villages comprising Rwebishekye parish participated in the training and planning for the census, mobilization activities and the health campaign (see Table 1). Throughout the census and the mobilization activities, the elected leaders remained engaged in pre-campaign activities. Elected leader census activities included organizing and overseeing the volunteer health teams (VHTs) during the census implementation, as well as performing door-to-door enumeration themselves. They also performed mobilization activities to promote campaign attendance including door-to-door visits to discuss the health campaign, church and mosque announcements, distribution of posters, and radio announcements.

**Table 1.** Feasibility metrics and outcomes during implementation of a community-led health campaign in rural Uganda

Community actor	Feasibility measure	CL-HC feasibility study intervention	Outcome
Elected village leaders	Elected leader acceptance & participation in training for pre-campaign and campaign activities	Monthly training led by SEARCH Trial consultants.	All village leaders (N = 8) accepted and participated in training.
Elected village leaders	Implementation of pre-campaign community mobilization activities	Peer-to-peer training by elected village leaders from a neighbouring parish that had previously participated in a SEARCH health campaign.	All village leaders designed and implemented community mobilization activities including: CHC poster placement throughout villages Announcements at places of worship Radio announcements Door-to-door discussions to promote CHC/answer questions within each leader’s village
Elected village leaders	Implementation of pre-campaign census enumeration	Training by SEARCH consultants and peer-to-peer training by elected village leaders from a neighbouring parish that had previously participated in a SEARCH study census.	All village leaders implemented a door-to-door census, enumerating 5,202 residents over two weeks in August 2014.
Local Ministry of Health clinic staff	Implementation of health screening services, including POC HIV, hypertension, diabetes and malaria screening	Working with clinic director to identify staff with screening service training that could participate in CL-HC.	Active, daily participation by local clinic staff during each of the six CLHC days. MoH clinic provisions (HIV test kits, blood pressure cuffs, etc.) leveraged for CLHC screening
Community residents	Participation in CLHC health services	Pre-campaign mobilization activities by locally elected leaders.	2,753 community residents (53%) participated in 6-day CLHC. HIV testing uptake by 1,474/1,584 (93%) adult campaign participants
Local Ministry of Health clinic staff	Measurement of uptake of health screening services, and community testing coverage	Training by SEARCH consultants.	CLHC participants successfully linked back to census enumeration, allowing for measure of campaign coverage Accountability and measurement of all services delivered with logbooks

Of 29 MoH-funded clinic healthcare workers at Bwizibwera Health Centre, 20 participated in campaign service provision throughout the CLHC, while the other nine remained at the health facility to perform ongoing, routine clinic activities and to accept referrals from the CL-HC. MoH clinic healthcare workers provided all healthcare service delivery, including point-of-care laboratory testing, and were present for the full duration of activities on all six days of the CLHC (see Table 1).

Specific feasibility challenges when working with locally elected leaders, VHTs and MoH health clinic workers included low literacy levels, which made name matching of census lists and campaign participants challenging due to misspellings, and generated occasional data entry errors when recording screening results. In addition, many of the VHTs and MoH workers anticipated additional payments for their work apart from the daily transport reimbursements and per diems (described above), respectively, on campaign days.

### Census

Out of an estimated 6,400 residents in the parish based on national census projections, local leaders enumerated a total of 5,202 residents in two weeks; 78% of the projected population. The census enumerated 2,162 children (<15 years), and 3,016 adults (≥15 years); 24 residents were enumerated without age information. Overall, 1,205 households were enumerated with an average household size of 3 persons (range: 1–15 persons/household).

### Community-led health campaign (CLHC)

Over six campaign days in September 2014, 2,753 (53%) residents participated in the CLHC, including 48% (1,037/2,162) of enumerated children and 53% (1,584/3,016) of adults; for 132 campaign participants’ age was not recorded. Among adult (≥15 years) campaign participants, 597/1,584 (38%) were men (Table 2), and 1,474/1,584 (93%) tested for HIV (Table 3). Adult HIV testing coverage was 40% (550/1387) among men residents, and 56% (916/1629) among women residents (eight adults with HIV test results had missing gender data). HIV prevalence was 105/1,474 (7.1%) among adults tested, and 11/645 (1.7%) among children tested. Per MoH clinic records, 17 of 116 (15%) HIV-infected persons were in HIV care at the local health centre; 83/99 (84%) newly identified HIV-infected persons were referred to care to the local health centre and accepted this referral; 16 of these lacked data on referral. Of 751 (27%) CLHC participants reporting fever, 24/428 (5.6%) of ≤10 year olds (a high-risk group for poor clinical outcomes) tested positive for malaria by RDT; 16/323 (5%) >10 year olds with fever tested positive for malaria. Of adults screened for hypertension, 271/1,452 (18.7%) had a blood pressure measure ≥140 mmHg systolic or ≥90 mmHg diastolic: 21.5% and 17% of men and women screened, respectively, had an elevated blood pressure measure (Table 3). Among adults screened for diabetes, 18/637 (2.8%) had a random blood sugar ≥11.1 mmol/L: 2.4% and 2.9% of men and women screened, respectively (Table 3). All participants who screened positive for hypertension or diabetes were referred to BWB HCIV for further confirmation and treatment.

**Table 2.** Characteristics of census-enumerated residents and community-led campaign participants, and the proportion of the census-enumerated population covered by campaign activities

Characteristics	All census-enumerated parish residents	Community led health campaign participants	% Population Covered
Total	**N (%)**	**N (%)**	**%**
	5202	2753	53%
Age^a^			
0–14 years	2162 (42)	1037 (38)	48%
15–50 years	2650 (51)	1288 (47)	49%
>50 years	366 (7)	296 (11)	81%
Gender			
Female, <15	1100 (21)	512 (19)	47%
Male, <15	1062 (20)	525 (19)	49%
Female, ≥15	1629 (31)	987 (36)	61%
Male, ≥15	1387 (27)	597 (22)	43%

^a^24 census-enumerated residents were missing age data, and 132 CLHC participants were missing age data.

**Table 3.** Community-led multi-disease health campaign services provided and screening results during a six-day campaign in rural Uganda

*Community Health Campaign Participants N = 2,753*	N	%
**HIV tested**	2,119	77%
**HIV positive**		
Children (<15 years) (N = 585 tested)	9	1.5%
Adult (≥15 years) (N = 1,474 tested)	105	7.1%
Age not recorded (N = 60 tested)	2	3.3%
Males (N = 865 tested)	36	4.2%
Females (N = 1240 tested)	76	6.1%
Gender unknown (N = 14 tested)	4	28.5%
**Malaria** (N = 2,753 screened)		
Self-reported fever	751	27.3%
Confirmed malaria, if febrile (N = 751 tested)	40	5.3%
Age≤10 years (N = 428)	24	5.6%
Age>10 years (N = 295)	12	4.1%
Age unknown (N = 28)	4	14.3%
**Hypertension, adults** (N = 1,452 screened)		
Systolic ≥140 or Diastolic ≥90 mmHg	271	18.7%
**Males (N = 540)**	116	21.5%
**Females (N = 912)**	155	17%
**Diabetes, adults** (N = 665screened)		
Random blood glucose ≥11.1 mmol/L	18	2.8%
**Males (N = 249)**	6	2.4%
**Females (N = 416)**	12	2.9%
**Urgent care** (N = 2,753)	107	3.9%
***Children services***		
**Vitamin A supplementation**		
6–12 months (N = 54)	33	61%
12 months-5 years (N = 420)	419	99.7%
**Mebendazole** (N = 698 with age ≤5 years)	543	78%
***Referrals***		
**Family planning** (FP; N = 900 women ≥18 years)		
Total number accepted same-day referral for FP	25	2.8%
Number received implants	23	2.6%
Number received IUD	2	0.2%
**Safe male circumcision** (N = 805 males ≥10 years)	91	11.3%

Out of 900 women (≥18 years) participating in family planning education at the CLHC, 25 (2.8%) accepted same-day referral to the local clinic for long-term family planning services and received either an IUD (n = 2) or an implant (n = 23); 20/25 (80%) women who accepted FP interventions were <32 years of age. Of 805 men and boys >10 years old evaluated for safe and voluntary, medical male circumcision, 91 (11%) accepted same-day referral to the local clinic for circumcision. 107/2,753 (3%) participants presented for urgent care evaluation and received treatment for their presenting complaints at the CLHC: those that required care that could not be addressed at the CLHC were referred to the health centre for further management.

### Census and campaign costs

The total operating cost of providing the census, mobilization activities, and all campaign services, including SEARCH consultation (i.e. two SEARCH trial study coordinators, administrative staff and one-time capital goods cost per day based on 5-year use), was $23,596.56 (2014 US dollars); an average of $8.57 per participant (Table 4). SEARCH consultation costs totalled $6,582.49, and included consultant staff salaries, data entry costs for analysis, costing activities, and car hires for transportation of SEARCH consultants to the Rwebishekye villages. Without these SEARCH-related expenses, the total cost was $6.18 per participant. The total cost per HIV+ person identified was $146.67/person. For a scaled-up replication model, we projected estimated costs using MoH facilities, capital goods and government staff average salaries, without SEARCH trial support, to be $16,328.20 total or $5.93 per participant in attendance.

**Table 4.** Overall costs and costs scaled-up to reflect Ministry of Health standard rates using a community-led health campaign approach

	Observed Census and Campaign		Scaled-up replication
Category of expenses	Overall costs (incl. SEARCH supported)	SEARCH- supported	Overall costs
Personnel (i.e. coordinator, counsellors, lab tech)	$6,337.84	$3,402.40	$944.34
Goods (i.e. HIV tests, drug treatments, timers, flyers)	$12,741.76	$2,138.97	$12,552.16
Services (i.e. transportation and fuel, catered meals)	$4,291.31	$815.47	$2,639.46
Capital Goods (i.e. laptops, tents, chairs)	$225.65	$225.65	$192.24
**Total**	**$23,596.56**	**$6,582.49**	**$16,328.20**

## Discussion

In this study, a health campaign planned by local village leaders and implemented by local clinic healthcare workers was able to reach over half of residents in a rural Ugandan community of over 5,000 persons within one month of planning and six days of implementation. Multi-disease health campaigns have been successfully implemented in sub-Saharan Africa and represent an effective strategy for rapid population-wide scale-up of HIV testing in rural, resource-limited settings [3–5]. However, increasing HIV testing coverage on a country level is likely to require both buy-in from local leaders and leveraging of existing clinical resources. In this feasibility demonstration project, we found that local leaders and clinic staff could successfully plan and implement a health campaign. In addition, by calculating cost per campaign participant, our data provide policy-makers and community leaders with estimates of the resources needed to expand multi-disease health campaigns across rural Africa. Importantly, village leaders from a neighbouring community that had performed similar campaigns in the past served as the primary consultants for campaign activities, suggesting a role for sequential community-to-community capacity building for this public health approach to large-scale HIV testing.

Community involvement in health campaign service delivery in resource-limited settings is likely to have a positive impact on health-seeking practices by local residents, as a community-led approach allows local leaders and clinic staff to exercise choice in selecting services that match residents’ health priorities. The importance of community involvement among other factors has also been demonstrated in community-based health campaigns that have not included HIV testing, including vaccination campaigns and bed net distribution, with or without the bundling of health services [12–14]. In Rwebishekye parish, these services included non-communicable disease (NCD), family planning and safe male circumcision services. We identified a high burden of undiagnosed diseases during the CLHC, even though multi-disease diagnostic capacity existed prior to the campaign at a nearby, local clinic, suggesting that the community health campaign approach reduced structural barriers (such as transport costs [15,16]) and increased access to and awareness of health services for local villagers. The level of resident participation among adults (53%) was lower than overall coverage reported from multi-disease campaigns carried out in a research setting such as the SEARCH trial (i.e. 71% overall coverage of HIV testing at campaigns across 32 communities). However, the SEARCH trial testing intervention also included home-based testing of campaign non-participants to achieve testing coverage targets, the feasibility of which also merits investigation outside of a research setting [5]. Heterogeneous service coverage across communities could also be due to differential trust in locally elected leaders, VHTs and/or clinic staff [17]. Uptake also varied by type of health service offered, with relatively low uptake of long-term family planning (2.8%) and medical male circumcision (11%) by campaign participants. This may reflect relatively lower demand for these services in this population or possibly concerns regarding stigma or opportunity costs associated with same-day transportation to the local clinic from a campaign site.

To our knowledge, our study is among the first to demonstrate the feasibility of conducting a rapid census and multi-disease health campaign, led and implemented by locally elected leaders and VHTs and local MoH clinic staff, respectively. Conducting a pre-campaign census serves a dual purpose of mobilizing residents to participate in the campaign while publicly demonstrating local leader support for the campaign, and providing a direct measure of the proportion of the community covered by campaign activities. This latter measure of coverage may serve as an indicator of the campaign’s success, thereby providing direct feedback to locally elected leaders and MoH clinic healthcare workers on which demographics they are failing to reach in their communities. The feasibility of a local census also suggests that a community-led hybrid approach to out-of-facility HIV testing, in which home-based testing of campaign non-participants is implemented after the campaign to maximize community coverage [5], may be feasible as well. Last, having local MoH clinic healthcare workers provide services within rural villages may provide an opportunity for increased clinic-community interaction and awareness of services available at local clinics, and merits further study.

The cost estimate of this multi-disease community-led campaign (including census enumeration and SEARCH support) of $8.57 per participant (2014 USD), is comparable to other community-based HIV testing approaches [18,19], and lower than the $11 per participant cost for similar multi-disease services conducted by SEARCH under a research setting in an adjacent community [7]. The lower cost in this CLHC may be a result of the low cost of leveraging available MoH clinic workers to provide services at the campaign. Furthermore, we estimate that in a scalable model in which local MoH-salaried staff replace all consultants, the CLHC could achieve still lower costs per participant (as low as $5.93). In addition, per person CLHC costs will vary with the number of campaign participants in attendance. The number of staff conducting this CLHC was chosen to accommodate 100% of community members accessing the services; had a greater proportion of the Rwebishekye community members identified in the census attended the campaign, the scale-up replication cost could have been lower.

This study has several limitations. First, our study may have overestimated campaign coverage if some residents were missed during the census. Additionally, name matching of the enumerated villagers and CLHC attendees was challenging due to variable spelling of names and multiple names provided by individuals. Biometric fingerprint matching is a more accurate method of identification and could be added into future CLHCs to address this challenge [5]. Second, costs in this study are based on estimated salaries and existing infrastructure and resources available from the Bwizibwera Health Centre IV and Rwebishekye community; these costs may differ by country and community. Third, newly identified HIV-infected persons may not reflect true new diagnoses as some of these persons may have been in care elsewhere, or may have known their HIV status but not yet engaged in care. Fourth, although the intervention was community led, it received guidance from consultants from the SEARCH trial and from a group of leaders from a neighbouring community with health campaign experience, which may have facilitated implementation and acceptance among the elected leaders in this community. Finally, linkage to care for those who screened positive for HIV, hypertension and diabetes was not measured. The focus of this study was the feasibility of implementing a community-led health campaign. However, all persons screening positive for these conditions at the CL-HC received prompt referral to the local clinic where treatment for HIV, hypertension and diabetes is available. We have previously reported linkage to care rates for HIV, hypertension and diabetes from similar campaigns in a neighbouring community [7].

## Conclusions

In summary, this community-led health campaign demonstrates the feasibility of having community leaders and health facilities plan and conduct a rapid census enumeration, mobilization activities, and a health campaign, with consultative support. With this community-led approach, over half of community residents participated in testing for HIV, while accessing other preventive health and treatment services. These data suggest that a community-led approach to multi-disease health campaigns is feasible, and could serve as a platform for rapid scale-up of out-of-facility HIV testing in a rural African setting.
